# Supramolecular peptide and protein systems – from design to application

**DOI:** 10.1039/d6ma90081j

**Published:** 2026-10-28

**Authors:** Ana M. Garcia, Jacek K. Wychowaniec, Ivan R. Sasselli, Verónica I. Dodero, Daniela Kalafatovic

**Affiliations:** a Facultad de Ciencias y Tecnologías Químicas, Instituto Regional de Investigación Científica Aplicada (IRICA), Universidad de Castilla-La Mancha Ciudad Real 13071 Spain; b AO Research Institute Davos Clavadelerstrasse 8 Davos 7270 Switzerland; c SDU Biotechnology, Department of Green Technology, Faculty of Engineering, University of Southern Denmark Campusvej 55 5230 Odense Denmark; d Danish Institute of Advanced Studies (DIAS), University of Southern Denmark Campusvej 55 5230 Odense Denmark; e Centro de Física de Materiales (CFM-MPC), CSIC-EHU Donostia-San Sebastián 20018 Spain; f Fakultät für Chemie, Biophysikalische Chemie (PCIII) & Org. und Bioorganische Chemie (OCIII), Universität Bielefeld Bielefeld 33615 Germany; g University of Rijeka, Faculty of Engineering Vukovarska 58 Rijeka 51000 Croatia daniela.kalafatovic@uniri.hr

## Abstract

Schematic development of supramolecular peptide and protein systems and assessing their design rules for real-world applications. Created in BioRender. V. Dodero (2026) https://BioRender.com/sl8atjb.
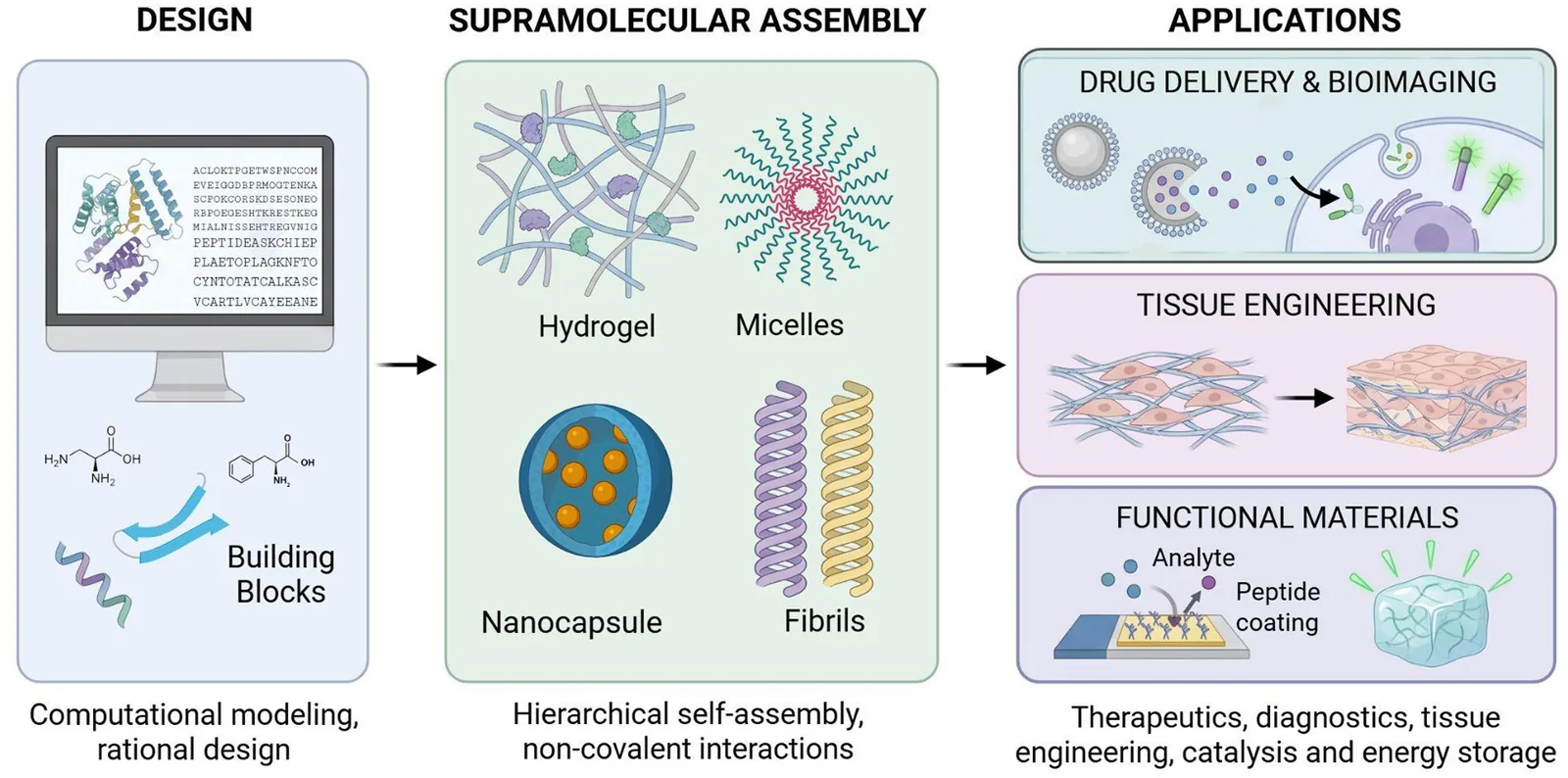

## Introduction to supramolecular peptide and protein systems

Supramolecular peptide and protein systems offer highly versatile platforms in which sequence, experimental conditions and non-covalent interactions direct the formation of organised supramolecular structures with different architectures showing tunable properties and functions. Developed in connection with CA23111 COST Action SNOOPY (Searching for nanostructured or pore forming peptides for therapy), this themed collection brings together recent advances in the design, synthesis, characterisation, and functional evaluation of peptide- and protein-based assemblies. The 15 research contributions and 1 review cover complementary levels of investigation, from fundamental studies of self-assembly, energy landscapes and structure–property relationships to dynamic soft materials, hybrid structures and protein nanocompartments. They also demonstrate the expanding application of these systems in catalysis, energy storage, regenerative medicine, drug delivery, immunotherapy and diagnostics, illustrating how molecular-level control can be translated into functional behaviour across multiple length scales. By combining experimental, computational and translational perspectives, the collection reflects both the interdisciplinary nature of the field and the scientific themes addressed within the SNOOPY network. Together, these studies connect fundamental understanding with emerging applications and provide a broad view of the current opportunities and challenges in the rational development of supramolecular peptide and protein materials.

## From molecular design to self-assembly pathways

A central theme of the collection is the need to understand how molecular information is translated from sequence to supramolecular organisation and ultimately into material properties. This challenge can increasingly be addressed before experimental synthesis through the integration of generative modelling, protein-structure prediction and molecular simulation. Lu and Buehler demonstrate this approach for spider-silk proteins by combining sequences generated using SilkomeGPT with OmegaFold structure prediction and molecular-dynamics simulations of unfolding. Their framework provides a computationally efficient route for exploring sequence-structure-mechanics relationships and identifying candidate proteins with tailored nanomechanical behaviour.^1^

Complementing this, several contributions experimentally dissect the factors that determine the outcome of peptide assembly. Castelletto *et al.* examine alternating tryptophan–arginine (WR) sequences and reveal a length- and pH-dependent balance between liquid–liquid phase separation and β-sheet fibrilization. Shorter WR peptides undergo self-coacervation under basic conditions, whereas increasing the number of repeats favours extended fibrils and nanotapes. If concentration is sufficiently high, the longer sequences also generate fibrillar hydrogels.^2^ This dependence on molecular design and environmental conditions is further developed by Kariuki, Rho and Perrier, who use time-resolved asymmetric-flow field-flow fractionation (AF4) to map the energy landscape of cyclic peptide–polymer conjugates. Their results distinguish a thermodynamically favoured hydrogen-bonding pathway leading to nanotubes from a competing hydrophobically driven route that produces metastable, disordered aggregates, demonstrating that preparation can be as important as molecular structure in determining the final assembly.^3^

Chemical modification offers another route for directing assembly. Shekhar *et al.* use carbamylation to neutralise the side-chain charges of lysine-repeat peptide amphiphiles, converting otherwise non-assembling polylysine derivatives into β-sheet-rich fibrillar nanostructures. By varying both peptide length and the length of the hydrophobic tail, the authors show that the number of lysine residues and the hydrophobic balance jointly regulate nucleation, aggregation kinetics and fibril morphology. The study illustrates how a post-translational modification associated with pathological protein aggregation can be repurposed as a design tool for functional peptide materials.^4^

The conventional relationship between hydrophobicity and short-peptide self-assembly is further challenged by the bioinspired hexapeptide Ac-SYTVTT-NH_2_, derived from type II cytoskeletal cochleal keratin. Despite its highly polar and protic sequence, the fully capped peptide self-assembles under physiological conditions into β-sheet-rich nanofibres that form stable hydrogels. Comparison with three terminally modified analogues highlights the decisive role of end-group chemistry in gelation, supported by simulations linking reduced hydration with stronger peptide–peptide association. The resulting hydrogels are mechanically stable, proteolytically degradable and cytocompatible, supporting fibroblast adhesion and spreading. This work therefore expands the design space of minimal peptide hydrogelators beyond conventional hydrophobic or aromatic motifs and highlights their potential for tissue-engineering applications.^5^ A complementary strategy is explored by Grelich-Mucha *et al.*, who selectively disrupt the hydrogen-bonding network of the amyloidogenic Aβ(35-42) fragment through a single amide-to-ester substitution. While the native peptide rapidly forms amyloid fibrils and the corresponding depsipeptide predominantly produces spherical aggregates, both retain substantial β-sheet character and display comparable intrinsic fluorescence. The strong pH dependence of this autofluorescence supports a mechanism involving proton transfer between peptide termini, indicating that long-range fibrillar order is not essential for the optical response and thus providing new insight into the molecular origin of amyloid autofluorescence.^6^

## Protein nanocages or similar systems

Protein-based nanocompartments provide a complementary route to controlling biochemical function through spatial organization and molecular confinement. In this collection, Pećanac *et al.* report the structural elucidation and engineering of bacterial encapsulins, revealing how these robust protein nanocages can serve as biocatalytic nanoreactors. Cryo-EM analysis of two newly identified encapsulins uncovered distinct architectures and demonstrated their ability to encapsulate active enzymes, enhance resistance to proteolytic degradation and regulate substrate transport through engineered pore structures, highlighting the potential of protein nanocompartments as tunable platforms for biocatalysis and biotechnology.^7^ Complementing this, Ghini *et al.* employed site-specific ^19^F NMR spectroscopy to probe the binding of gold-based metallodrugs to ferritin nanocages. By linking spectroscopic signatures to local conformational changes through molecular dynamics simulations, the study provides atomistic insight into protein–metal interactions and demonstrates how ferritin-based nanocarriers can be interrogated at the molecular level to support the rational development of drug delivery systems.^8^

## Responsive/hybrid peptide materials for soft matter, catalysis and energy

Moving from assembly mechanisms to macroscopic materials, some works in this collection highlight how reversible interactions and molecular architecture can be used to regulate mechanics and responsiveness. Murphy *et al.* synthesise complementary aldehyde- and acyl-hydrazide-functionalised polypeptides with linear and star-shaped topologies, which form dynamic hydrogels through Schiff-base crosslinking. Mixing different topological architectures provides access to different mechanical properties while retaining rapid self-recovery and self-healing. Importantly, the gels support human mesenchymal stem-cell viability and increasing metabolic activity over 14 days, connecting network design with biological performance.^9^

Responsive behaviour also emerges from the association of polypeptides with functional inorganic components. Kuroiwa *et al.* co-assemble a redox-active pentanuclear iron complex with amphiphilic poly(l-aspartic acid)-*block*-poly(l-leucine) copolypeptides, thereby overcoming the poor aqueous solubility of the metal cluster. Changes in pH control both peptide conformation and morphology, producing transitions between multilayered vesicle-like structures and extended sheets while simultaneously modifying the oxidation state and electrochemical response of the Fe_5_ complex. This work establishes a strategy for creating artificial metalloprotein-like environments, where peptide organisation regulates water accessibility, ion transport and electron transfer.^10^

The ability of peptide assemblies to influence electronic and ionic transport is extended towards energy storage by Alemán and his team. Neutral and protonated α/β-dipeptides are self-assembled between PEDOT layers to generate multilayer pseudocapacitor electrodes. The protonated TFA-DP1 assemblies provide both aromatic pathways for electronic transport and mobile counterions for ionic transport, acting as dielectric elements within submicrometric PEDOT layers, demonstrating that short peptide assemblies can serve as active components in biodegradable electronic devices.^11^

## Biomedical applications, regenerative materials, and diagnostics

The translational potential of supramolecular peptide- and protein-based materials is clearly reflected in applications spanning regenerative medicine, immunotherapy and diagnostics. In regenerative medicine, Lababidi *et al.* provide preclinical evidence that 6% *Saussure costus*-loaded polycaprolactone-gelatin electrospun nanofibers could function as an antimicrobial and wound-healing dressing. The nanofibrous scaffolds combined favourable mechanical properties, sustained release of bioactive compounds from the extract of this medicinal plant against *Staphylococcus aureus*, *Pseudomonas aeruginosa*, and *Escherichia coli* and biocompatibility on human model cells. Furthermore, authors showed *in vivo*, an enhanced wound closure, collagen deposition and tissue regeneration, highlighting the potential of hybrid protein based-biomaterial systems for skin repair.^12^ Complementing these advances, Celikkin *et al.* developed a form of methacrylate pepsin digested pulmonary extracellular matrix (dECMMA) that was crosslinked to gelatin methacrylate (GelMA) for the formulation of a composite peptide-biomaterial ink, that enables the 3D printing of vascular constructs. The dECMMA/GelMA composite led to enhanced endothelialization, endothelial network formation and pro-angiogenic activity, demonstrating how extracellular-peptide matrix-derived biomolecular cues can support the fabrication of vascularised tissues for regenerative applications.^13^ Immunotherapeutic strategies are represented by the work of Seetharaman *et al.*, who incorporated cervical tumour cell-derived antigens into thermostable nanoarchaeosomes, made of 80% SOPC conventional phospholipid and 20% archaeal lipids to create an efficient protein-based therapeutic nanovaccine. These nanoarchaeosomes promoted antigen-presenting cell maturation, stimulated T-cell proliferation and cytokine production, and elicited tumour-cell killing, highlighting the potential of protein-loaded nanostructures as platforms for cancer immunotherapy.^14^ In the area of diagnostics, Sabaine *et al.* developed peptide-functionalised gold nanoparticle biosensors based on short synthetic peptides from the SARS-CoV-2 Spike protein. It was demonstrated that the peptide sequence strongly affects sensor performance by combining surface-enhanced Raman spectroscopy and electrochemical impedance spectroscopy. In conclusion, the effective, sensitive and variant-specific detection of anti-SARS-CoV-2 antibodies was shown, illustrating the utility of peptide-based molecular recognition, for next-generation biosensing technologies.^15^ Complementing these experimental advances, the accompanying review on triple-negative breast cancer surveys emerging nanoscale theranostic approaches that integrate targeted drug delivery, molecular imaging and precision oncology, while also addressing key mechanisms of tumor progression and metastasis, providing a broader perspective on the expanding role of nanoscale biomaterials in cancer diagnosis and treatment.^16^

## Summary and future outlook

Collectively, the studies in this themed collection illustrate a field that is rapidly progressing from fundamental molecular peptide and protein design towards application-driven materials and technologies. Advances in computational design, mechanistic understanding of self-assembly, and high-resolution structural characterization are increasingly enabling the rational engineering of peptide- and protein-based systems with predictable properties and functions. At the same time, emerging applications in catalysis, energy storage, regenerative medicine, immunotherapy and diagnostics demonstrate the growing translational potential of these materials. Looking forward, the integration of experimental, computational and bioengineering approaches will be essential for bridging molecular-scale understanding with real-world performance, enabling the development of adaptive, multifunctional and clinically relevant supramolecular peptide and protein systems. Together, these efforts position peptide and protein fields to address key challenges in healthcare, sustainability and advanced materials science, while continuing to expand the scope and impact of biomolecular self-assembly. Such progress is further strengthened by collaborative European networking initiatives, including COST Actions such as SNOOPY, which foster interdisciplinary collaboration, knowledge exchange and the coordinated development of shared methods, standards, and best practices.

## References

[cit1] Lu W., Buehler M. J. (2025). Generative design and molecular mechanics characterization of silk proteins based on unfolding behavior. Mater. Adv..

[cit2] Castelletto V., de Mello L., Seitsonen J., Hamley I. W. (2025). Fibrillization *versus* coacervation in arginine–tryptophan repeat peptides: effects of sequence length and pH. Mater. Adv..

[cit3] Kariuki M., Rho J. Y., Perrier S. (2025). Mapping the energy landscape of a supramolecular system *via* time-resolved asymmetric-flow field flow fractionation. Mater. Adv..

[cit4] Shekhar V., Paul T., Gadhavi J., Gupta S. (2025). Self-assembly of carbamylated lysine repeat peptide amphiphiles into fibrillar nanostructures. Mater. Adv..

[cit5] Novo P., Sánchez-Temprano A., Gauci V., Lopez-Blanco R., Sánchez-Fernández R., Rodríguez-Trillo A., Marchetti A., Fasoli E., Jimenez-Millan S., Sasselli I. R., Pigliacelli C., Metrangolo P., Fernandez-Trillo P., Pazos E. (2026). A bioinspired highly polar hexapeptide forms β-sheet nanofibers and cell-adhesive hydrogels. Mater. Adv..

[cit6] Grelich-Mucha M., Garcia A. M., Torbeev V., Olesiak-Bańska J. (2026). The influence of H-bonding network disruption on morphology, structure and autofluorescence of amyloids. Mater. Adv..

[cit7] Pećanac O., Belyy A., Martin C., Kleissen R., Fraaije M. W., Lončar N. (2025). Biocatalytic application and structural elucidation of robust bacterial protein nanocages. Mater. Adv..

[cit8] Ghini V., Di Paco G., Cosottini L., Rosato A., Turano P. (2025). ^19^F NMR as a tool to probe drug binding and structural dynamics in ferritin-based nanocarriers. Mater. Adv..

[cit9] Murphy R. D., Lobianco F., Chiaradia V., Galindo J. M., Hodgkinson T., Heise A. (2026). The influence of polymer topology and side chain functionality on the Schiff-base reactivity of biocompatible polypeptide hydrogels. Mater. Adv..

[cit10] Kuroiwa K., Tada T., Ichise M., Masaoka S. (2026). Supramolecular hybrid of Fe pentanuclear complex/diblock copolypeptide amphiphiles with pH-responsive nano/microstructures in water. Mater. Adv..

[cit11] Quintana-Romero D. A., Fontana-Escartín A., Gelmi M. L., Gul M., Pérez-Madrigal M. M., Bucci R., Alemán C. (2026). Self-assembled α/β-dipeptides as dielectrics to improve the behavior of multilayered conducting polymer pseudocapacitors. Mater. Adv..

[cit12] Lababidi J. M., Fytory M., Abouzid A. E., El-Qassas J., Gad A. T., Ahmed O. M., El-Badri N., El-Said Azzazy H. M. (2025). Promising wound healing activity of *Saussurea costus* loaded PCL–gelatin nanofibers. Mater. Adv..

[cit13] Celikkin N., Tirelli M. C., Maiullari F., Cisneros C. G., Kaushik V., Volpi M., Aerts A., Mohren R., Vandenbosch M., Cardinaels R., Mignon A., Costantini M. (2026). Methacrylated pulmonary dECM-enriched GelMA bioinks promote endothelialization and angiogenesis in 3D printed tubular constructs. Mater. Adv..

[cit14] Seetharaman A., Duraisamy P., Ariraman S., Ramanathan P., Sudhakar S. (2026). Nanoarchaeosomes loaded with tumor antigens elicit antigen-presenting cell activation and T cell response for cervical cancer immunotherapy. Mater. Adv..

[cit15] Sabaine A. E., Castro-Kochi A. C. H., Mancini R. S. N., Silva M. R. A., Sepulveda A. F., Oliveira J. R., Remuzgo C., Santos K. S., Oliveira V. L., Kochi L. T., Kubota L. T., Mamián-López M. B., Alves W. A. (2025). Peptide-based biosensors for variant-specific detection of SARS-CoV-2 antibodies. Mater. Adv..

[cit16] Sonalisha S., Kirti A., Asima S. P., Parashar R., Pal S., Barik D., Naser S. S., Souto E. B., Verma S. K. (2026). Translational paradigm of advanced nanoscale strategies for triple negative breast cancer (TNBC): mechanistic insights, metastatic pathways, and emerging theragnosis. Mater. Adv..

